# Biliary Calculi—Passage of a Large Number, and of Unusual Size

**Published:** 1871-10

**Authors:** N. S. Davis


### BILIARY CALCULI.—PASSAGE OF A LARGE NUMBER, AND OF UNUSUAL SIZE.

Case Reported to the Chicago Medical Society November 6th, 1871.

By N. S. DAVIS, M. D.

Mrs. B——, aged 55 years, was attacked on the 21st of August last with violent pain in the epigastrium, near the lower margin of the ribs, and over the region of the gall bladder. There was a little quickening of the pulse, but no fever. The extremities soon became cool, the pulse quick, but small and weak, and acute tenderness was manifested over the course of the hepatic ducts. I feared a supervention of peritoneal inflammation, but expected that the severity of the pain would abate after a few hours. Hydrate chloral was ordered to be given in large doses, together with some morphia; also active narcotic fomentations were applied over the abdomen.

None of these measures, however, were successful in overcoming the pain. Two or three evacuations occurred, which were thin and intermixed with mucus, but not of the clay color which would indicate a retention of the bile. The acute pain was overcome by keeping the patient stupefied with chloral, but for a week she remained in a critical condition. She then began to improve. The tenderness over the abdomen remained, however, with a slight tendency to diarrhœa, but hardly any evidence of jaundice.

On the 19th of September the patient was again attacked with the same symptoms, more violently than before. The warm fomentations were renewed, and chloral promptly given. She was also placed in a warm bath, and remained there until symptoms of faintness began to be manifested. On the second day a moderate laxative was given, and on examining the evacuations the calculi here exhibited were obtained. There are thirteen of them, varying from $\frac{2}{10}$ to $\frac{4}{10}$ of an inch in diameter. They are of rather irregular outline, and many of them having been apparently worn off at the corners, display very nicely the concentric layers of which they are composed.

After the passage of these, the patient began rapidly to recover, but at the end of a week there still remained some slight feeling of discomfort, nausea, etc., and the bowels continued irritable, but there were no signs of active inflammation. With a view of preventing the further formation of calculi, she was directed to take 6 gtts. nitric acid, well diluted with water, three times a day. This, however, being found to irritate the bowels, was withdrawn, and liquor potassa substituted in doses of 10 gtts., at first, and gradually increased to 15 gtts. To improve the condition of the bowels, she was also ordered some powders consisting of bismuth sub. nitras, 6 grs., lapulin, 1 gr., and morph. sulph., $\frac{1}{12}$ gr.

The patient soon afterward went East on a visit, but had three repetitions of the attacks during her absence; a number of calculi being passed on each occasion, amounting to about 60 in all. Since her return home, however, during the past few weeks she has remained perfectly well.

Another somewhat similar case occurred in my practice some six or seven years ago. A Jewish lady had been subject to repeated attacks of severe pain, etc. Being called in the midst of one of the paroxysms, I was satisfied from the character and situation of the pains, and from the marked jaundice, that it was a case of biliary calculi. The nitric acid was ordered for her, 6 gtts. to be taken at each meal time, and continued for several weeks. She had one subsequent attack, a short time after commencing treatment, but has had none since.

I can recollect three other cases where the re-formation of calculi has been entirely prevented. In two of the cases an opposite course of treatment was tried, an alkali, liquor potass, being given instead of the acid.

Some years ago, a member of this society related a case of biliary calculi cured by sweet oil, given in large doses. I tried the treatment in one case, and in the course of 48 hours a large amount of matter was passed, consisting of little balls of a light color, and varying in size from a pea up to a hickory-nut. On examination they proved, however, to be nothing but the solid stearine of the oil moulded into balls. I have also several times had specimens of supposed biliary calculi sent to me from the country, which, however, by the time that they reached me, had melted or re-dissolved into oil. True calculi, such as these before us, are not softened by oil.

Our aim in the treatment of these cases should be, first, to relieve temporarily the pain, by narcotics, warm fomentations, etc.; and secondly, to so change the constitution of the bile as to prevent the future formation of new calculi. Their formation is probably caused by an excess of cholesterine in the bile, which becomes crystallized. The calculi formed are sometimes so large as never to pass through the ducts; but being retained, and blocking up the ducts, they produce permanent derangement, congestion, atropy, jaundice, etc.

The treatment by ether, acids, or alkalies seems to accomplish the desired change in the constitution of the bile.

